# The Validation of Two-Dimensional and Three-Dimensional Radiographic Measurements of Host Bone Coverage in Total Hip Arthroplasty for Hip Dysplasia: A Comparison with Intra-Operative Measurements

**DOI:** 10.3390/jcm12196227

**Published:** 2023-09-27

**Authors:** Chang-Jin Yon, Kyung-Jae Lee, Byung-Chan Choi, Ho-Sung Suh, Byung-Woo Min

**Affiliations:** Department of Orthopaedic Surgery, School of Medicine & Institute for Medical Science, Keimyung University, Daegu 42601, Republic of Korea; poweryon88@gmail.com (C.-J.Y.); oslee@dsmc.or.kr (K.-J.L.); bcchoikr@dsmc.or.kr (B.-C.C.); 210515@dsmc.or.kr (H.-S.S.)

**Keywords:** computed tomography, hip dysplasia, host bone coverage, three-dimensional, total hip arthroplasty

## Abstract

Several methods have been introduced to measure the host bone coverage of the acetabular component after total hip arthroplasty (THA). The aims of this study were (1) to validate two-dimensional- and three-dimensional-based host bone coverage measurements by comparing intra-operative measurements, and (2) to determine the minimum host bone coverage for achieving stable cup fixation after THA in hip dysplasia. The clinical outcomes of each patient were evaluated during their final follow-up period using the Harris Hip score (HHS). The coverage of the host bone was analyzed by comparing 2D-based, 3D-based, and intraoperative assessments. The mean HHS was increased significantly from 60.84 ± 14.21 pre-operatively to 93.13 ± 4.59 (*p* < 0.0001). The host bone coverage ratio measured intraoperatively was 83.67 ± 3.40%, while the ratio measured by 3D CT reconstruction was 82.72 ± 3.59%. There was a strong positive correlation between the intra-operative host bone coverage and the 3D-based one (r = 0.826, *p* < 0.0001). It is recommended that 3D-based measurements are used to evaluate the host bone coverage after THA in patients with hip dysplasia. In addition, achieving a minimum host bone coverage of 75% is recommended for the attainment of stable cup fixation

## 1. Introduction

Hip dysplasia is a common cause of osteoarthritis (OA) in the hip. Various factors are associated with the development of hip dysplasia, including developmental dysplasia of the hip, pyogenic arthritis of the hip in childhood, Legg–Calve–Perthes disease, and multiple epiphyseal dysplasia [1,2,3,4]. Common features of hip dysplasia resulting from such conditions include a small, shallow acetabulum, along with an inadequate coverage of the femoral head [5]. Abnormal joint anatomy induces a stress concentration on the femoral head. Finally, early secondary hip OA can be developed in hip dysplasia [6].

Total hip arthroplasty (THA) is the preferred treatment for the advanced hip OA caused by hip dysplasia, and the number of THAs is annually growing [7]. One quarter of THAs performed in patients over 40 years of age were due to underlying hip dysplasia [6]. However, positioning the acetabular cup during the performance of THA is still challenging for the surgeon due to the shallow acetabulum and lack of acetabular coverage of the femoral head. In order to achieve a stable fixation of the acetabular cup and minimize complications such as recurrent dislocation [8] and osteolysis after THA [9,10,11], both an optimal cup orientation [11,12] and a sufficient bone coverage of the acetabular cup [13,14,15,16] are critical factors.

Various techniques have been proposed in an effort to improve the host bone coverage of the acetabular cup, including the use of a smaller cup, positioning at a high hip center [17], performing cotyloidplasty [15], and resorting to structural femoral autografts [13,14]. The intentional perforation of the medial wall of the acetabulum has been proposed as a useful method for facilitating the achievement of favorable outcomes for the insertion of a cementless acetabular cup. Multiple studies have compared the effects of controlled medial wall fractures and autografts from the bulk femoral head. Some of these studies have reported on the repercussions of a controlled fracture of the medial wall [16]. In addition, satisfying the mid-term results from impaction grafting for the correction of acetabular deficiency with more than 30% bone defects has been documented [18]. To overcome acetabular bone defects, several methods such as oblong cup, trabecular metal augmentation, and reinforced devices have also been suggested [19]. Despite efforts to reduce the failure rate, the burden of revision of overall THA is still from about 2.2% to 7.2% [7]. Furthermore, from 33% to 39% of hip dysplasia patients treated with THA required the revision of their acetabular cup [13,14,18] and from 5.9% to 15.9% of patients required re-operation after revision THA of their acetabular cup [19].

Several methods can be applied for the measurement of the host bone coverage of the acetabular cup after THA. Some researchers have proposed the use of 2D-based methods from pelvic plain radiographs, while others have recommended the use of 3D-based methods from computed tomography (CT) scans [20,21,22]. Some results have indicated inconsistencies between the 2D-based measurements from plain radiographs and the 3D-based measurements from CT scans [20,22]. Some studies have recommended use of 3D CT-based measurements for determining cup coverage, because radiograph-based measurements tend to overestimate the host bone coverage. Some of these studies have reported that the iliac oblique view is the most dependable 2D imaging modality for the assessment of cup coverage [21]. Several studies have reported that from 45% to 80% is the minimum required host bone coverage for the achievement of stable cup fixation [8,14,23,24]. However, these values are too broad to be applied during operations. There is currently neither a consensus about the minimum host bone coverage required for the achievement of stable cup fixation nor a gold standard method for assessing host bone coverage.

This study serves two purposes: (1) to validate 2D- and 3D-based host bone coverage measurements by comparing them with intra-operative measurements, and (2) to determine the minimum host bone coverage required for the achievement of stable cup fixation after THA for the treatment of hip dysplasia.

## 2. Materials and Methods

### 2.1. Study Cohort Enrollment

A total of 29 patients with 31 hips affected by hip OA resulting from hip dysplasia were enrolled in this retrospective study. Patients who underwent THA using cementless components between February 2017 and May 2018 at the authors’ institution were screened for eligibility. Hip dysplasia was defined as a decreased lateral center-edge angle (CEA) less than 20° and an increased Tonnis angle of more than 10°. Hip OA cases in which hip dysplasia was thought to be the main pathology were also included, although the lateral CEA and tonnis angle did not indicate hip dysplasia. It was confirmed by two senior hip surgeons (B-W. Min and K-J. Lee).

**Figure 1:**
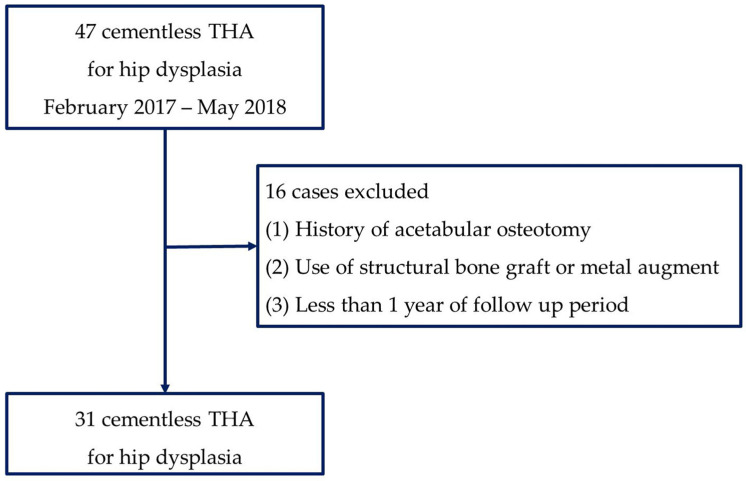
Flowchart of cohort enrollment.

During the study period, 43 patients with 47 hips affected by OA resulting from hip dysplasia underwent THA. The patients’ medical records, including their demographics and medical history, were evaluated. A total of 16 hips (14 patients) with a surgical history of acetabular osteotomy, the use of a structural bone graft, or metal augment for acetabular deficiency during THA, and those with a follow-up period of less than one year were excluded from the study. Thus, 31 hips (29 patients) were included in this study (Figure 1). All the patients underwent a post-operative 3D CT (Somatom Sensation 64, Siemens Healthineers, Munich, Germany) for an assessment of the cup orientation and a measurement of the host bone coverage. The study subjects included 4 (12.9%) male and 27 (87.1%) female patients. The mean age at the time of surgery was 63.0 ± 12.4 years. The patients were followed for an average of 54.9 ± 17.3 months (range: 17–72 months). The average height and weight of the patients were 152.5 ± 8.1cm and 59.3 ± 12.9 kg, respectively. The average lateral CEA was 1.96° with a standard deviation of 16.7° (range: −48.4 to 24.0°), while the Tonnis angle was 29.43° with a standard deviation of 6.4° (range: 14.5 to 40.5°). Of the 32 cases, 16 cases were categorized as the dysplastic type, 15 cases as the low-dislocation type, and one case as the high-dislocation type of hip dysplasia.

### 2.2. Surgical Procedure and Prosthesis

All THA procedures were conducted using the modified hardinge approach. The procedures were performed by a single surgeon at one institution. The acetabular cup was inserted with cementless press-fix fixation after the exposure of the subchondral bone, and a minimum host bone coverage of approximately 75% was achieved with adequate medicalization. Two or three supplemental acetabular screws were added. The consecutive next procedures were performed without delay. The intra-operative measurements were conducted in the labs while operating. The quantified intra-operative measurement of the host bone coverage was reported to the operator around the end of the procedure. Then, we could confirm the intra-operative host bone coverage without a prolonged operative time.

The Continuum (Zimmer-Biomet, Warsaw, IN, USA) acetabular component design was used for the implantation in all hips. The external diameter of the acetabular component ranged from 52 mm to 68 mm. An elevated liner was used in 13 hips, while the remaining 18 hips were fitted with a neutral liner. The Biolox Delta ceramic head was used in all the hip implants. For femoral implants, preoperative templating was used for the selection of the implant that best suited the morphological features of each patient’s medullary cavity (Table 1).

### 2.3. Clinical Outcomes

An evaluation of the clinical outcomes for each patient was performed using the Harris Hip Score (HHS) during the latest follow-up period. In addition, the incidence of complications after THA was evaluated during the follow-up period.

### 2.4. Cup Position and Fixation

**Figure 2:**
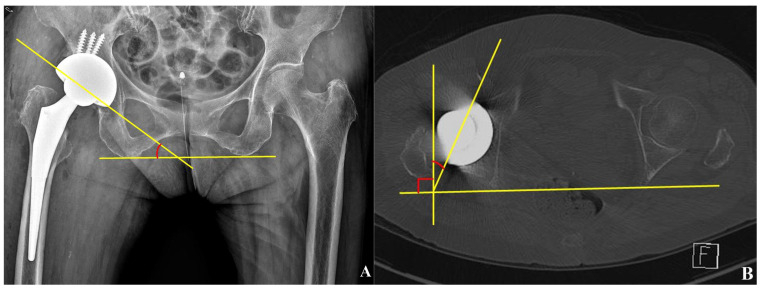
Measurement of cup inclination and anteversion. (**A**) Cup inclination measurement. The angle between the line tangential to the face of the acetabular cup and the line which is tangential to the inferior border of the both ischial tuberosity. (**B**) Cup anteversion measurement. The angle between the line tangential to the face of the acetabular cup and the line perpendicular to posterior pelvic margins at the level of the sciatic notch on axial CT scans.

The cup inclination was measured on anteroposterior radiographs of the pelvis as the angle between the line tangent to the face of the acetabular cup and the line tangent to the inferior border of both ischial tuberosities (Figure 2A). Cup anteversion was identified on an axial CT scan as the angle between the line tangent to the face of the acetabular cup and the line perpendicular to the posterior pelvic margins at the level of the sciatic notch (Figure 2B) [25]. The cup fixation status was evaluated by observing the cup migration in serial plain radiographs and the modified DeLee and Charnley classification system on last follow up plain radiograph. (Table 2) [20,24]. A rotational change of more than 5° or a linear change of more than 2 mm in the serial radiographs were considered as indications of cup migration [26].

### 2.5. 2D-Based Measurement of Host Bone Coverage from Plain Radiographs of the Pelvis

**Figure 3:**
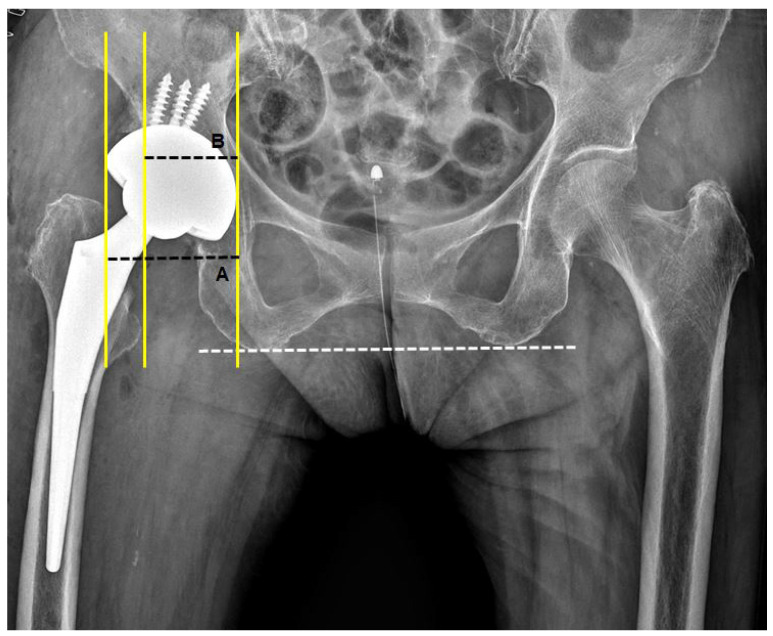
Measurement of bone coverage index (BCI). BCI was defined as the ratio of horizontal width of the covered portion of cup (B) to horizontal width of the whole cup (A).

The bone coverage index (BCI) was used for an evaluation of the 2D-based host bone coverage, measured on plain AP radiographs of the pelvis taken 2–4 days after surgery. The ratio of the horizontal width of the covered portion of the cup to the horizontal width of the entire cup was calculated for the determination of the BCI (Figure 3).

### 2.6. 3D-Based Measurement of Host Bone Coverage from CT Scans

**Figure 4:**
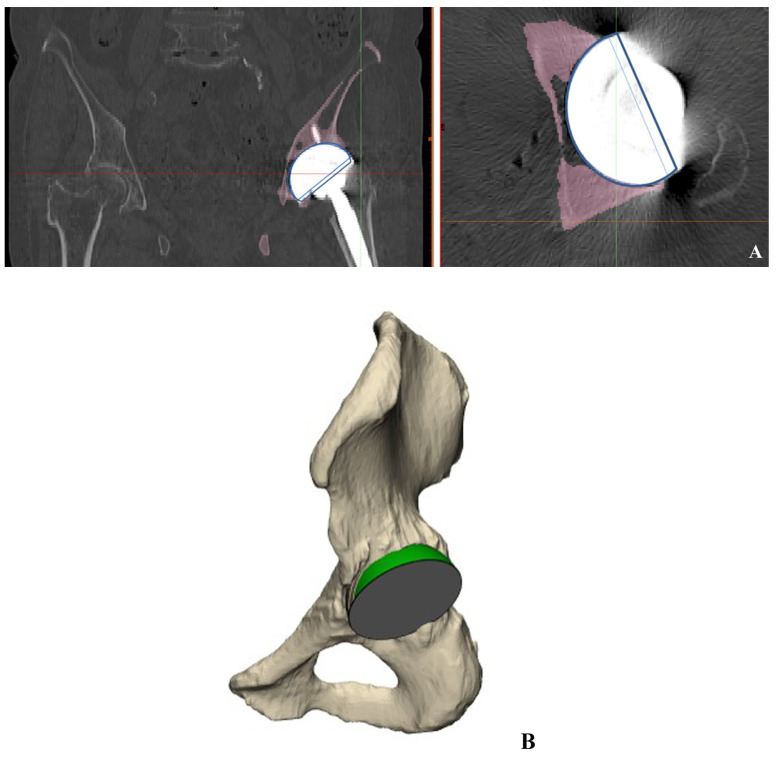

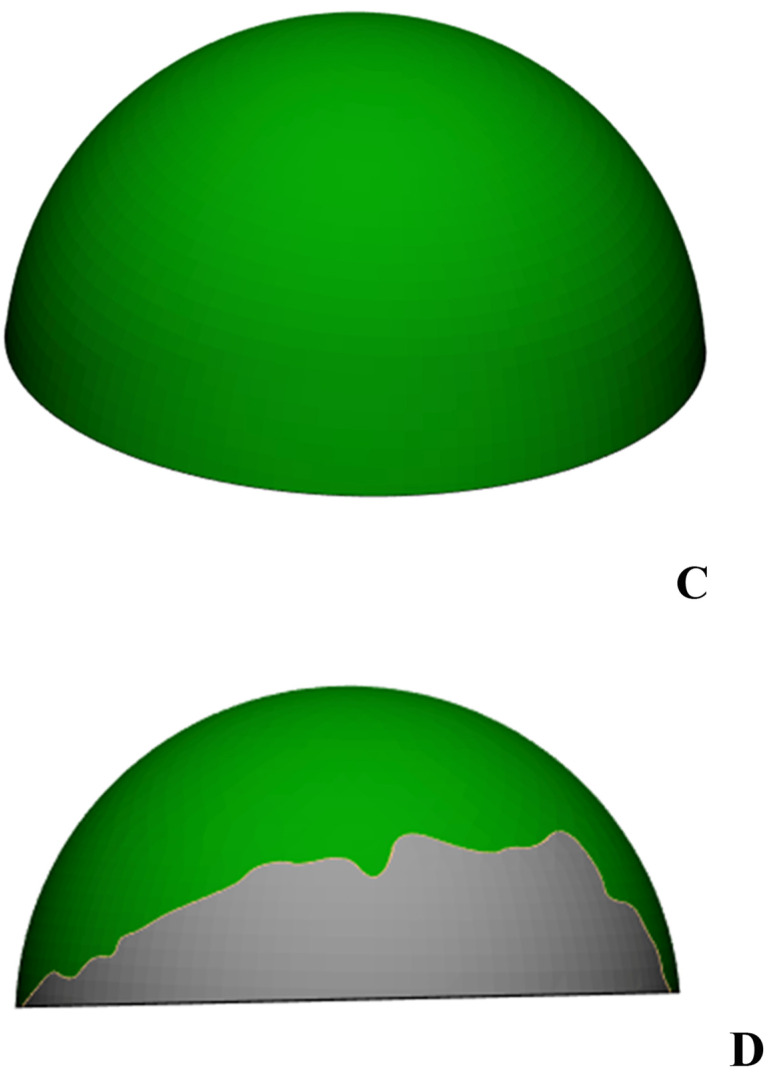
Measurement of 3D CT-based host bone coverage. (**A**) Recognize and cluster the serial outlines of pelvic bone and acetabular component structure. (**B**) Surface reconstruction images were achieved. (**C**,**D**) After reconstruction of the 3D image, the whole surface of acetabular cup and uncovered surface of cup was calculated.

Axial CT scans were used to obtain images of the host bone coverage for the 3D reconstruction. Multislice CT images with multiplanar reconstruction were also acquired. Data from the CT scanning of the pelvis were stored in DICOM (Digital Imaging and Communications in Medicine) format and were used by the computer-aided design (CAD) software, known as Mimics version 19 (Materialise, Leuven, Belgium), for the 3D reconstruction. Brightness was used in Mimics for the identification and grouping of the serial outlines of the pelvic bone and the components of the acetabular structure (Figure 4A). Surface reconstruction images were obtained simultaneously after stacking the categorized outlines (Figure 4B). The concurrently built surface models were automatically partitioned into independent models. Following the reconstruction of the 3D image, the calculation of the total surface area of the acetabular cup and the exposed part of the cup was performed (Figure 4C,D). The uncovered surface, as calculated, was subtracted from the entire surface area of the acetabular cup for the calculation of 3D CT-based host bone coverage.

### 2.7. Measurement of Intra-Operative Host Bone Coverage

**Figure 5:**
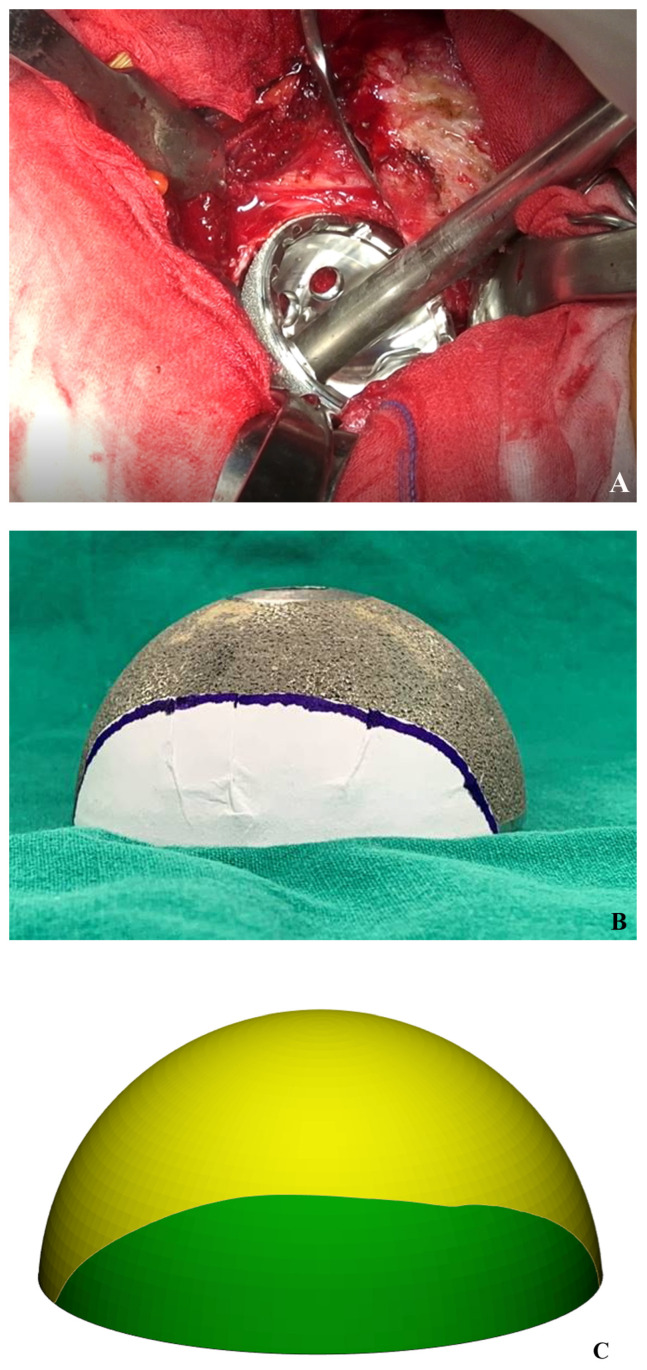
Measurement of intra-operative host bone coverage. (**A**) Press-fit fixation of acetabular cup, uncovered surface was recognized. (**B**) Marking on same size of cup trial. (**C**) Surface area of both whole surface of acetabular cup and uncovered surface of cup measured during operation.

In this study, the surface area of the uncovered surface of the acetabular cup was measured after press-fit fixation. A marking of the same size was made on the cup trial during the performance of surgery on all the hips included in the study (Figure 5A,B). The Mimics software was used for the computation of the surface area of both the entire surface and the uncovered surface of the acetabular cup, which were measured during the performance of surgery after scanning the paper showing the measurements (Figure 5C).

### 2.8. Statistical Analyses

All the statistical analyses were performed using the IBM SPSS Statistics software (version 26.0; IBM Corp., Armonk, NY, USA). The intraclass correlation coefficient (ICC) test was used to demonstrate the similarity of the measurement results with the use of the two methods. A paired *t*-test was used for a comparison of the pre-operative clinical score with the score from the last follow-up period. Pearson correlation coefficient was used to examine the correlation between the two methods. A correlation coefficient (r) of <0.20 was regarded as no correlation, 0.20 ≤ r < 0.40 as poor, 0.40 ≤ r < 0.6 as fair, 0.6 ≤ r < 0.8 as good, and 0.8 ≤ r as an excellent correlation. A *p*-value of <0.05 was considered to be statistically significant.

## 3. Results

### 3.1. Clinical Outcomes

The average preoperative Harris Hip Score (HHS) was 60.84 ± 14.21 (range: 25–80), which improved to 93.13 ± 4.59 (range: 82–99) at the last follow-up. This increase in the HHS was statistically significant. None of the cases required a prosthesis revision for any reason (*p* < 0.0001). In addition, there were no cases of a recurrent dislocation or infection in the study cohort.

### 3.2. Cup Position and Fixation

The average cup inclination was 35.3° ± 7.3°, with a range from 25.2° to 47.6°, and the average cup anteversion was 23.8° ± 12.8°, with a range from −14.1° to 46.1°. The average number of supplementary screws was 2.5; two screws were used in 15 hips and three screws were used in 16 hips.

Cup migration was not observed in all cases. All the cups showed a stable fixation, as demonstrated by the bone ingrowth over a postoperative period of five years. Out of the hips examined, 27 hips were classified as type 1A, three hips as type 1B, and one hip as type 1C. There were no cases with radiologic evidence of osteolysis or implant loosening (Table 3).

### 3.3. Correlation of Three Methods of Host Bone Coverage

**Figure 6:**
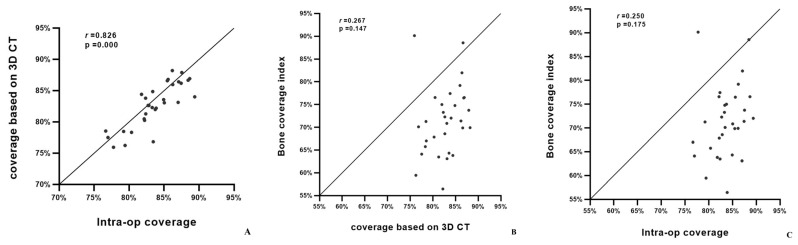
Scatter plot of BCI, 3D CT-based, intra-operative host bone coverage. (**A**) Correlation between the intra-operative host bone coverage measurement and 3D-based measurement from CT scans. (**B**) Correlation between 3D-based measurement from CT scans and BCI from plain radiographs. (**C**) Correlation between the intra-operative host bone coverage measurement and BCI from plain radiographs.

The average BCI was 71.46 ± 7.53% (range 56.46–90.12%). The mean intra-operative bone coverage was 83.67 ± 3.40% (range 76.67–89.38%), while the 3D-based coverage was 82.72 ± 3.59% (range 75.95–88.22%). A robust positive correlation was observed between the intra-operative measurement of the bone coverage and the 3D-based measurement from the CT scans (r = 0.826, *p* < 0.0001). No statistically significant correlations were observed between the BCI and other two methods (BCI and 3D; r = 0.267 *p* = 0.147, BCI and intra-op; r = 0.250 *p* = 0.175) (Figure 6A–C). The intra- and interobserver reliability were excellent (intraclass coefficient correlation = 0.987, *p* < 0.0001).

## 4. Discussion

**Figure 7:**
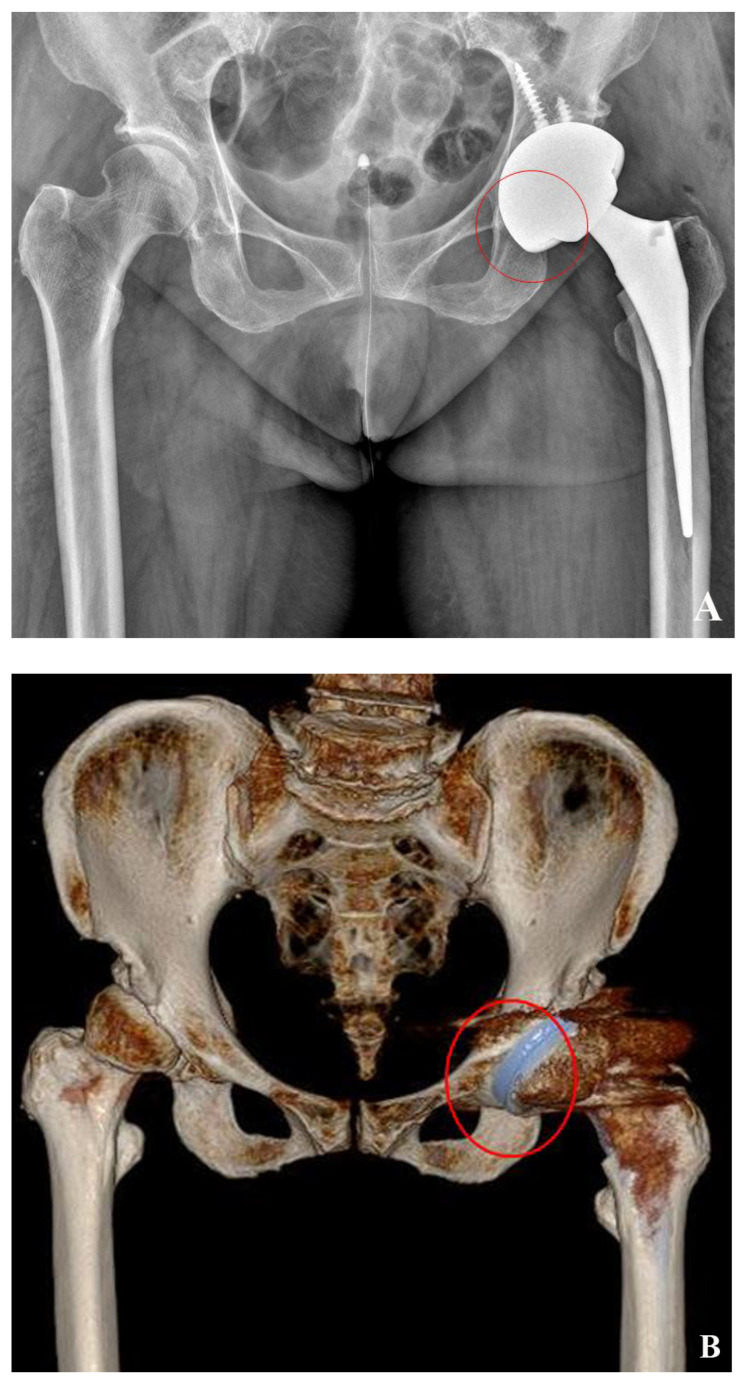
Differences of host bone coverage between plain radiograph and 3D CTs. Inferomedial side of acetabular cup (red circle) was calculated as covered surface in 2D-based host bone coverage measurement. (**A**) On the other hand, the lack of bone coverage on the inferomedial side of the cup (red circle) was detected in the 3D reconstruction of the CT scan (**B**).

The findings of this study demonstrated a strong correlation between the intra-operative measurements and the 3D-based measurements of the host bone coverage from the CT scans. The 2D-based measurements from the plain radiographs underestimated the host bone coverage when compared to the other two methods used in our study. Because the BCI was measured on a 2D projected image of an AP radiograph of the pelvis, the contact surface between the host bone and the acetabular cup was projected as a curved line when using 2D-based measurements. These differences may explain why no correlation was observed between the BCI and other methods of measurement. We made an effort to visualize the whole acetabular component using the modified hardinge approach. As a result, we could consider the uncovered area of the inferomedial side of the acetabular cup during the operation. The lack of bone coverage on the inferomedial side of the cup could be detected in the 3D reconstruction of the CT scan. In contrast, calculations could only be performed for the superolateral side of the uncovered surface of the cup in the 2D-based measurements of the host bone coverage. (Figure 7A,B). The surface of the acetabular cup covered by osteophyte was included as coverage in the 3D-based and intra-operative measurements, but it was excluded in the 2D-based measurements. We believe that the discrepancy between the 2D-based, 3D-based, and intra-operative measurements might have been a result of the technical differences between these methods. Despite these differences, we have to question whether these surfaces really affected the stability of the cup. Finally, further studies about practical host bone coverage, which really contributes to stability, are needed.

Optimal cup positioning is challenging due to the small, shallow acetabulum in patients with hip dysplasia. Several methods can be applied for an assessment of the acetabular cup position based on radiographs of the pelvis and CT scans. Numerous studies have suggested an acceptable range for acetabular cup positioning [25]. A consensus has been reached with regard to the safe zone for cup inclination and anteversion. On the other hand, neither consistent outcomes nor a gold standard have been reported for the evaluation of host bone coverage, which is also an essential factor in achieving a stable cup fixation in the management of hip dysplasia [27]. There are various outcomes with regard to the correlation of 2D with 3D CT-based measurements of host bone coverage, and there is a broad range of required host bone coverage. A coverage of a minimum from 45% to 80% of the host bone is required to achieve the stable fixation of the acetabular cup [8,14,23,24]. In our study, all the acetabular cups were stably fixed without cup migration on a serial radiograph, and stable bone ingrowth was finally observed in all cases. The results of the intra-operative and 3D CT-based measurements showed that a minimum host bone coverage of 75% was sufficient. We recommend a minimum host bone coverage of 75% in order to achieve stable cup fixation with bone ingrowth at a mean follow up period of 54.9 months. However, this does not mean that a host bone coverage of less than 75% is insufficient. Conducting additional studies will be needed in order to demonstrate the minimum host bone coverage required for the achievement of stable cup fixation.

In addition, limited research on intraoperative measurements of host bone coverage has been reported. To the best of our knowledge, only two studies have reported on intraoperative measurements of host bone coverage. Yong et al. proposed the use of bone wax for the measurement of intraoperative host bone coverage. Bone wax was applied to the exposed surface of the acetabular cup, and a quantification of the bone wax surface was subsequently performed using 3D scanning. A comparison of the radiographic cup coverage with the intraoperative host bone coverage showed a significant correlation between the two [28]. Xu J. et al. suggested a positive linear relationship between the base and height of the uncovered portion of the 3D cup and its uncovered area. Based on these findings, they determined that ensuring a 3D host bone coverage of 75% is possible when the height of the uncovered portion of the 3D cup measured intra-operatively is not more than 0.5 times the diameter of the cup. Finally, the height of the uncovered portion could be a useful intra-operative index for assisting surgeons in the assessment of the host bone coverage [22]. The outcomes of the two studies were not identical. In our study, no significant correlation was observed between the radiograph-based measurements of the host bone coverage and host bone coverage measured intra-operatively. This result is consistent with the result of the latter study, although the intra-operative measurements were similar to those reported in the former study. The differences in the measurements of the host bone coverage could be interpreted as the reason for the discrepant results. Finally, conducting additional studies will be needed for an examination of intra-operative measurements of host bone coverage.

Several principles must be followed for the achievement of a stable cup fixation in patients with hip dysplasia [29,30,31]. First, ensuring the sufficient contact of the acetabular cup and medial host bone through adequate medialization with a 1 mm or 2 mm press-fit fixation of the hemispherical acetabular cup is important in order to overcome the challenge posed by the shallow cotyloid fossa and small acetabulum of patients with hip dysplasia. Second, several studies have already reported satisfactory results with the use of the press-fit fixation of a cementless hemispherical cup. However, despite the ongoing controversy regarding the effectiveness of additional screw fixation in many studies reported in the literature [31,32], we believe that it can be helpful in augmenting the stability of cups in cases of hip dysplasia. Screws are not commonly used for acetabular cups during the performance of THA in a normal acetabulum. However, we attempted to insert screws along the longest possible trajectory in cases of hip dysplasia. Although one screw was inserted into the posterosuperior osteophyte of three patients in this study, the purchasing power of the screw was sufficient. We believe that screw insertion is an effective method for fixing acetabular cups.

Our study has several acknowledged limitations. First, this study had a retrospective design without a control group, and the number of included cases was low, meaning that the cohort could only represent a minority of patients with hip dysplasia. A lower limit of host bone coverage for stable cup fixation could not be suggested in our study. Second, the follow-up duration was short. Thus, long-term outcomes might not be accurately reflected. Nevertheless, many studies have demonstrated that early cup migration is associated with the late aseptic loosening of prostheses [26,33]. Based on these outcomes, we expect good long-term outcomes for stably fixed acetabular cups, even though the follow-up duration was short in this study. In the future, there may be a need to conduct well-designed studies to evaluate the usefulness of intra-operative measurements of host bone coverage, as well as large-scale prospective studies for the assessment of host bone coverage. In addition, the mimics program used in this study has not been extensively utilized. From this perspective, it can be concluded that the practicality of this study is unsatisfactory. The use of an intuitive and accurate method for the evaluation of host bone coverage in the operating room would be a more practical approach.

However, to the best of our knowledge, this is the first study comparing three methods for measuring host bone coverage based on three distinct modalities. In addition, a stable fixation of the cup was accomplished in all cases using our intraoperative technique for the measurement of host bone coverage. We believe that the findings of this study will be very helpful for surgeons performing THAs on patients with osteoarthritis of the hip caused by hip dysplasia.

## 5. Conclusions

Intraoperative measurements of host bone coverage show a high degree of correlation with measurements of host bone coverage using 3D CT scans. Therefore, it is believed that both methods can be considered as reliable for the evaluation of host bone coverage. However, measurements based on radiographs did not show a correlation with the other methods. We recommend the use of intraoperative and 3D CT-based measurements for the assessment of host bone coverage after THA in patients with hip dysplasia. In addition, achieving a minimum host bone coverage of 75% is recommended for the attainment of a stable cup fixation.

## Figures and Tables

**Table 1:** Demographic and prosthesis data.

Demographics	
Age at surgery (years)	63.0 ± 12.4 (range: 30–81)
Follow up period (months)	54.9 ± 1.4 (range: 17–72)
Height (cm)	152.5 ± 8.1
Weight (kg)	59.3 ± 12.9
Pre-op radiologic parameters	
Lateral CEA	1.96 ± 16.7 (range: −48.4–24.0)
Tonnis angle	29.43 ± 6.4 (range: 14.5–40.5)
Crowe type	
Dysplastic	16 (51.6%)
Low dislocation	14 (45.2%)
High dislocation	1 (3.2%)
Prosthesis data	
Acetabular cup size	(52/54/56/58/60/62/68) 1/2/7/13/4/3/1
Supplementary screw (#2/#3)	16/15
Liner (neutral/elevated)	18/13
Head (32 mm/36 mm)	8/23

Values are shown as mean ± standard deviation (range) unless otherwise indicated. CEA: center edge angle.

**Table 2:** Modified DeLee and Charnley classification system.

Fixation Grade		Definition
Stable bone ingrowth	IA	No radiolucent line
	IB	Radiolucent line in 1 zone
	IC	Radiolucent line in 2 zones
Stable fibrous fixation	II	Complete radiolucent line, width < 2 mm
Unstable fibrous fixation	III	Complete radiolucent line, width ≥ 2 mm, or Cup migration

**Table 3:** Clinical and radiologic outcomes.

Parameters	Outcomes
Clinical outcomes	
Pre-op HHS	60.84 ± 14.2 (range 25–80)
Post-op HHS	93.13 ± 4.6 (range 82–99)
Recurrent dislocation	0
Periprosthetic joint infection	0
Radiologic outcomes	
Cup inclination (°)	35.3° ± 7.3 (range 25.2–47.6)
Cup anteversion (°)	23.8° ± 12.8 (range −14.1–46.1)
Modified DeLee and Chanley classification of fixation	
I (A/B/C)	27/3/1
II	0
III	0

## Data Availability

Data are available on request to the corresponding author, who must previously consult the institution’s ethics committee.
